# Development and optimization of the Suna trap as a tool for mosquito monitoring and control

**DOI:** 10.1186/1475-2875-13-257

**Published:** 2014-07-07

**Authors:** Alexandra Hiscox, Bruno Otieno, Anthony Kibet, Collins K Mweresa, Philemon Omusula, Martin Geier, Andreas Rose, Wolfgang R Mukabana, Willem Takken

**Affiliations:** 1 Laboratory of Entomology, Wageningen University and Research Centre, Wageningen, The Netherlands; 2 International Centre of Insect Physiology and Ecology (icipe), Nairobi, Kenya; 3 Biogents AG, Regensburg, Germany; 4 School of Biological Sciences, University of Nairobi, Nairobi, Kenya

**Keywords:** *Anopheles* mosquito, Malaria vector, Suna trap, CDC light trap, MM-X trap, Attractant, Odour bait, Surveillance

## Abstract

**Background:**

Monitoring of malaria vector populations provides information about disease transmission risk, as well as measures of the effectiveness of vector control. The Suna trap is introduced and evaluated with regard to its potential as a new, standardized, odour-baited tool for mosquito monitoring and control.

**Methods:**

Dual-choice experiments with female *Anopheles gambiae* sensu lato in a laboratory room and semi-field enclosure, were used to compare catch rates of odour-baited Suna traps and MM-X traps. The relative performance of the Suna trap, CDC light trap and MM-X trap as monitoring tools was assessed inside a human-occupied experimental hut in a semi-field enclosure. Use of the Suna trap as a tool to prevent mosquito house entry was also evaluated in the semi-field enclosure. The optimal hanging height of Suna traps was determined by placing traps at heights ranging from 15 to 105 cm above ground outside houses in western Kenya.

**Results:**

In the laboratory the mean proportion of *An. gambiae* s.l. caught in the Suna trap was 3.2 times greater than the MM-X trap (P < 0.001), but the traps performed equally in semi-field conditions (P = 0.615). As a monitoring tool , the Suna trap outperformed an unlit CDC light trap (P < 0.001), but trap performance was equal when the CDC light trap was illuminated (P = 0.127). Suspending a Suna trap outside an experimental hut reduced entry rates by 32.8% (P < 0.001). Under field conditions, suspending the trap at 30 cm above ground resulted in the greatest catch sizes (mean 25.8 *An. gambiae* s.l. per trap night).

**Conclusions:**

The performance of the Suna trap equals that of the CDC light trap and MM-X trap when used to sample *An. gambiae* inside a human-occupied house under semi-field conditions. The trap is effective in sampling mosquitoes outside houses in the field, and the use of a synthetic blend of attractants negates the requirement of a human bait. Hanging a Suna trap outside a house can reduce *An. gambiae* house entry and its use as a novel tool for reducing malaria transmission risk will be evaluated in peri-domestic settings in sub-Saharan Africa.

## Background

Effective monitoring of mosquito populations should provide information regarding the possible transmission intensity of mosquito-borne diseases in a given area at a particular time. Understanding vector population dynamics could provide early warning for an outbreak of disease or act as an outcome measure in evaluating the impact of a vector control programme [1,2].

An ideal monitoring tool would sample without bias; i.e. the trap would collect mosquitoes representative of all species, ages and gonotrophic stages found in the environment of the trap. For the widest possible application the tool would be effective wherever human hosts are found, which may include domestic and peri-domestic environments in urban as well as rural settings, inside and outside houses. The trap would also be effective during the times of day when vectors are most active. Medical entomologists work towards the development of traps that meet as many of these requirements as possible [3-5].

Monitoring of malaria mosquitoes poses a number of specific challenges; during its lifecycle a mosquito will pass through both aquatic and terrestrial life stages, and different species may occupy vastly differing ecological niches [6-9]. The most efficient malaria vectors are closely associated with the domestic environment and bite humans in and around their dwellings, whilst other species are predominantly found outdoors and may feed on both humans and animals [10-12].

The feeding habits of mosquitoes may be exploited for surveillance purposes. Thus, methods have been developed to intercept mosquitoes while they are host seeking. Historically, the most commonly used tool for monitoring malaria vectors was the human landing catch (HLC). This approach is considered to provide the best measure of man-biting rate and peak time of biting in a specific time and place [13-17]. Given the potential for exposure of volunteers to infective mosquito bites, as well as non-standardized trapping due to variation between the attractiveness of volunteers, and their ability to catch mosquitoes in the process of biting, an effective non-biased mosquito sampling tool, such as a trap, is needed.

Mechanical traps have been developed to substitute for the HLC [4]. Such traps may contain attractive stimuli that lure mosquitoes to the collection site. Because CO_2_ acts as a universal mosquito attractant, many traps are baited with this chemical [18]. For more selective mosquito species, additional volatile chemicals are added to the bait, significantly enhancing the catch [19-21].

At present, the most widely used trap for mosquito monitoring in and around the domestic environment is the CDC light trap (CDC LT) (John W. Hock company, Gainesville, FL). The CDC LT is generally used indoors, positioned beside a human-occupied bed net, without the addition of a synthetic chemical lure. Mosquitoes that are attracted to, but unable to reach, the human inside the bed net are caught when they fly around the trap [22,23]. The requirement for positioning the trap close to a human host means that the CDC LT is mainly limited to use indoors and is not an effective tool for monitoring the outdoor-biting population [23]. Studies comparing the performance of CDC LTs against HLC have reported widely variable results from different geographic locations [14,16,24,25].

Whilst the CDC LT has remained largely unchanged since the 1970s, the past decades have led to an increased understanding of which organic volatiles stimulate host-seeking mosquitoes to fly towards a host [26-28]. Traps baited with these odours have since formed an increasingly useful tool for monitoring mosquito populations. The Mosquito Magnet-X^®^ (MM-X) trap (American Biophysics corporation, North Kingstown, RI) is an odour-baited mosquito trap, which may be used outdoors or indoors [29-31] with mosquitoes attracted to a plume of odours and CO_2_ dispensed from the trap. Once a mosquito is close to the source of the odours, it is sucked into the trap by a counter-flow ventilation system [32]. Another odour-baited trap is the BG-Sentinel (Biogents AG, Germany) [33]. This suction trap disperses odours over a large surface, thus imitating convection currents of a host. Although widely used for dengue transmitting mosquitoes, the potential of the trap for collecting vectors of malaria has been recently explored [34].

A novel trap for malaria mosquito monitoring and control has been developed in collaboration between Biogents AG (Regensburg, Germany), the International Centre of Insect Physiology and Ecology (Mbita Point, Kenya) and the Laboratory of Entomology at Wageningen University and Research Centre (Wageningen, The Netherlands). The Suna trap is a new modification of the BG-Sentinel trap, but uses the same patented trapping technology. This paper describes the results of laboratory, semi-field and field studies to evaluate the performance of this new trap against the MM-X trap and the widely used CDC LT.

As well as using traps for mosquito monitoring, it has been suggested that an effective trap could also be used as a tool for mosquito control when used in combination with existing methods such as bed nets [35]. By daily removal trapping of mosquitoes, the size of a local vector population could be diminished, thus people would be exposed to fewer potentially infective bites. A lower entomological inoculation rate (EIR) would reduce the force of infection, bringing about a reduction in the basic reproductive number for a disease (R_0_) and possible elimination of a disease if R_0_ is brought below 1 [1].

The aims of this study were to evaluate the relative trapping efficacy of the Suna trap, CDC LT and MM-X trap, and to assess whether mosquito house entry could be reduced by hanging a Suna trap outside a traditional mud-walled African house. Experiments were conducted in laboratory, semi-field and field conditions.

## Methods

### Study sites

The studies described here took place in the behavioural room of the Laboratory of Entomology at Wageningen University and Research Centre (The Netherlands), semi-field screenhouses and the MalariaSphere at the Thomas Odhiambo Campus (TOC) of *icipe* (International Centre of Insect Physiology and Ecology, Mbita Point, western Kenya) (00°25’S, 34°13’E) and in field conditions at Ahero, western Kenya (00°34’S, 034°65’ E).

The behavioural room at Wageningen University measures 3.8 m long, 3.7 m wide, 3.2 m high and contains a large netted chamber measuring 3 m in length, 2.5 m in width and 2.5 m in height (see Additional file 1 for layout diagram). A 15-Watt bulb directed upwards towards a white cotton sheet on the ceiling provided low-lux lighting (artificial moonlight).

Dual choice experiments in semi-field conditions in Kenya were conducted in screen-walled greenhouses (“screenhouses”) [36]. In these structures the glass had been removed from the walls and replaced by gauze. A large mosquito-netting cage (length 11 m, width 7 m, height 2.5 m, with a 3 mm mesh-width) was suspended inside the screenhouse. The sand floor was watered daily in order to maintain high humidity levels.

Comparisons between the CDC LT, MM-X trap and Suna trap took place in the MalariaSphere [37] (see Additional file 2 for layout diagram). Briefly, a mud-walled, grass-thatched house was constructed in a screenhouse containing grasses, crops and other plants found growing in the local area (plantain banana, wild sage, black-jack, *Parthenium* weed, castor bean, Guinea grass and Napier grass) to mimic the conditions around a house in a typical Luo village of the area surrounding Lake Victoria in Kenya. Inside the house was a bed covered by an untreated bed net, allowing a human volunteer to sleep in the house at night. Whilst experiments were taking place, the door of the house was closed, thus the open eaves were the only house entry point for mosquitoes.

Field experiments to determine optimal height positioning of the Suna trap took place at Kigoche village in Kisumu County, western Kenya. Traps were suspended from overhanging roofs of mud-walled, metal-roofed houses with open eaves. Houses had not received indoor residual spraying (IRS) within the past six months and were located within 100 m of rice fields that were flooded during the time of the study (August-September 2012). Annual rainfall in this area ranges from 1000- 1800 mm, temperatures fall between 17 - 32°C and relative humidity is between 44 and 80%. Each house was occupied during the study with residents sleeping under insecticide-treated bed nets, as is standard practice for this region, and as advised by the Kenyan Ministry of Health.

### Mosquitoes

The *Anopheles gambiae sensu stricto (s.s.)* mosquitoes used for laboratory experiments originated from Suakoko, Liberia and have been cultured at the Laboratory of Entomology (Wageningen, The Netherlands) since 1987. The colony is of the *An. gambiae s.s.* M form and is hereafter referred to as *Anopheles coluzzii*. The mosquitoes were reared in climate-controlled rooms at 27 ± 1°C, 80 ± 5% relative humidity, with a 12-hour light: 12-hour dark cycle. Adults emerged from pupae into gauze-covered cages measuring 30 × 30 × 30 cm. Within the cages adults had access to a 6% (w/v) glucose solution provided on filter paper. Females were blood fed daily and provided with moist filter paper on which to oviposit. Eggs were placed in tap water in plastic trays containing Liquifry No.1 fish food (Interpet Ltd., United Kingdom), larvae were fed Tetramin^®^ baby fish food (Melle, Germany) and pupae were collected and allowed to emerge into BugDorm cages (MegaView Science, Taiwan).

The *An. gambiae s.s.* colony used during semi-field experiments in Kenya originated at Mbita Point and has been cultured at the *icipe-*TOC since 2001. The colony is of the *An. gambiae s.s.* S form and is hereafter referred to as *An. gambiae*. Larval rearing in Kenya took place under ambient conditions in screen-walled greenhouses. Pupae were collected on a daily basis and placed in cages measuring 30 × 30 × 30 cm to emerge. Once emerged, adults had access to 6% glucose (w/v) solution provided on filter paper and were blood fed three times a week. Eggs were laid on filter paper then transferred to plastic trays with filtered water from Lake Victoria. Emerged larvae were fed daily on Tetramin^®^ baby fish food (Melle, Germany).

Adult females used in laboratory and field experiments were between five and eight days old, had never received a blood meal and were starved by providing access to only distilled water on cotton wool for 10–18 hours before experiments took place.

### The Suna trap

**Figure 1:**
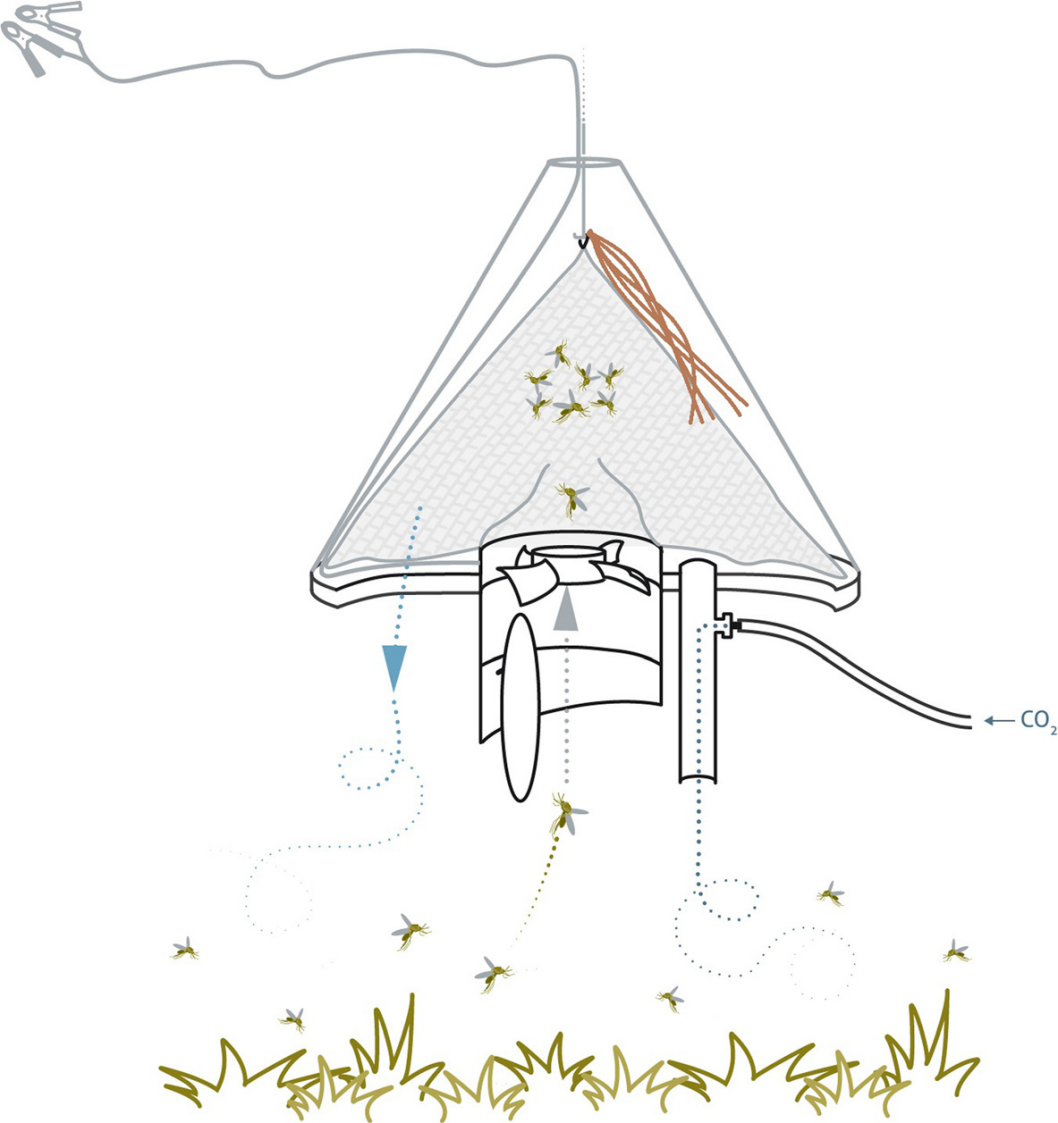
**Cross-sectional schematic view of the Suna trap.** When connected to a power supply the ventilator rotates and generates airflow under the cover of the trap. The air moves around an odour-bait (here shown as brown strips of nylon), which is suspended inside the trap between the catch bag and plastic trap cover. The air becomes saturated with odour that is attractive to mosquitoes. Air plumes leave the trap through holes in the trap base (blue arrow) and mosquitoes fly towards this attractive cloud of odour, as well as a plume of CO_2_, which is released through a pipe in the trap base. Mosquitoes that come close to the trap are sucked into the trap through the funnel and ventilator (grey arrow) and are captured in a bag where they die due to dehydration.

The Suna trap, named after the Dholuo word for mosquito, consists of five main components (Figure 1); a funnel and ventilator section, carbon dioxide release pipe, perforated plastic base, netting catch bag, hanging tripod and conical plastic cover. When the trap is connected to a 12 volt power supply the ventilator rotates, sucking air up through the funnel at a rate of 3.1 m/s, thus opening the funnel shutter gate. As air circulates under the conical cover of the trap, volatiles from a synthetic chemical blend of attractants are released from the nylon strips suspended from the hanging tripod. The odour-saturated air is forced out of the trap through holes in the plastic base at a rate of 0.5 m/s. This generates a flow of attractants, which are carried away from the trap. In addition, a plume of CO_2_ diffuses from the CO_2_ release pipe, mimicking breath of a host. In effect, the combination of odours and CO_2_ forms a human surrogate.

Mosquitoes encountering these odours fly upwind towards the trap and, when they are in close proximity to the funnel, they are sucked into the trap through the ventilator. Inside the trap they are contained in the catch bag. When the power supply is turned off, the shutter gate automatically drops to a closed position due to a weighting mechanism and mosquitoes are unable to escape. Mosquitoes caught inside the trap die due to dehydration and lack of food.

### Comparison between the Suna trap and the MM-X trap in laboratory and semi-field conditions

In the behavioural room in The Netherlands and in a semi-field screenhouse in Kenya, similar experimental setups were used to carry out a dual-choice experiment comparing mosquito trapping efficacy of the Suna trap against the MM-X trap (see Additional file 1). During any given replicate of the experiment, one Suna trap and one MM-X trap were suspended simultaneously in opposite corners of either the behavioural room or the screenhouse. Suna traps were suspended at 30 cm above the ground, as demonstrated to be the most effective height for this trap during field experiments. MM-X traps were suspended at 15 cm above ground level [29]. The positions of the traps were alternated for each experimental replicate and mosquitoes were released from a point equidistant from the two traps. In The Netherlands and Kenya, both traps were baited with a blend of ammonia, L-lactic acid, tetradecanoic acid, 3-methyl-1-butanol and 1-butylamine [38]. In the behavioural room, pressurized CO_2_ was supplied from a cylinder at 250 cc/min. In the semi-field setup, CO_2_ was produced through a yeast and molasses fermentation process (250 ml molasses, 17.5 g yeast, 2 litres water), shaken vigorously for 30 seconds [39].

In The Netherlands, 50 unfed female *An. coluzzii* were used for each experimental replicate and experiments were carried out during the mosquito dark photoperiod (artificially set between 00:00 h and 12:00 h) with a duration of one hour per replicate. In Kenya, 200 unfed female *An. gambiae* were released into the screenhouse at 20:00 h and the experiment was stopped at 06:30 h the following morning.

At the end of all laboratory and semi-field experiments traps were placed in a freezer at -20°C in order to knock down mosquitoes for counting. Temperature and relative humidity were measured during all experimental replicates using a Tinytag^®^ Ultra data logger (model TGU-1500, INTAB Benelux, The Netherlands). Any remaining mosquitoes died during the day because of exposure to high daytime temperatures and starvation.

### Comparison of the Suna trap, MM-X trap and CDC light trap under semi-field conditions

The trapping efficacy of the Suna trap was compared against that of the MM-X trap and CDC LT under semi-field conditions in the MalariaSphere in Kenya. Over the course of two experimental series, each of 24 nights duration (24 nights March – April 2013 and 24 nights February - March 2014), one trap per night was suspended next to the foot end of a untreated bed net, occupied by a male, aged 30 years, inside the MalariaSphere experimental hut (see Additional file 2). Trap models were allocated randomly over the 24 nights of each experimental series with each trap used eight times over the course of the experiment.

The Suna trap and the MM-X trap were baited with the MB5 blend of attractants [38], and were supplied with CO_2_ produced through a process of yeast and molasses fermentation, as described above. The CDC LT remained without odour bait, according to the standard protocol for the use of this trap [24]. During the first series of experiments the light in the CDC LT was turned off, but during the second series of experiments the light in the CDC LT was on. Traps were suspended at heights above the ground that have previously been demonstrated to be most effective for each trap. The Suna trap was positioned with the funnel opening at 30 cm above ground level (see results described in this paper), the MM-X trap was suspended 15 cm above the ground [29,31] and the CDC LT was suspended with the fan section at 50 cm above the ground [30].

Each night at 20:00 h, 200 unfed female *An. gambiae* were released into the MalariaSphere (50 from each of the four release points indicated in Additional file 2). Mosquitoes were released from four release points in order to simulate the possibility of mosquitoes approaching a house from multiple breeding sites under field conditions, and to avoid directional bias of mosquitoes accessing the hut when only one release point is used. The MalariaSphere remained closed until 06:30 h the following morning when the experiment was stopped. During each night of the study the temperature (°C), relative humidity (%) and total rainfall (mm) were recorded.

At the time of stopping the experiment, the number of females remaining in each of the release cups was counted. The number of females resting on the walls inside the house was counted as a measure of mosquitoes entering the house but not entering the trap.

### The performance of the Suna trap as a tool to reduce mosquito house entry in semi-field conditions

Measurement of a possible reduction in mosquito house entry that could be achieved by suspending a Suna trap outside a house was performed in the MalariaSphere, following a similar experimental setup as described in the previous experiment.

During each of 32 experimental nights 200 unfed female *An. gambiae* were released into the MalariaSphere at 20:00 h and experiments were concluded at 06:30 h the following morning. As in previous experiments, an adult male volunteer slept underneath an untreated mosquito net on a bed inside the MalariaSphere hut. On alternate nights a Suna trap was hung outside the house at 30 cm above ground level (see Additional file 2 for trap positioning). As described above, the trap was baited with the MB5 blend of attractants and CO_2_ produced by yeast and molasses fermentation. On subsequent nights no trap was hung outside the house. On all nights a CDC LT with the light off was hung inside the house at a height of 50 cm above ground, beside the foot end of the bed. The CDC LT was not baited with a synthetic lure, as is standard for the use of this trap.

During every night of the experiment, the number of mosquitoes captured inside the CDC LT was counted, as well as the number of females resting on the interior walls of the MalariaSphere house. This provided an estimate of the number of mosquitoes entering the house overnight. On nights when the Suna trap was positioned outside the house, the number of mosquitoes captured in this trap was also counted.

### Study to determine the optimal height positioning of the Suna trap under field conditions

A number of Suna trap prototypes were acquired in August 2012 and these traps were used to conduct a study into the optimal height positioning of the traps under field conditions. Eight houses were selected for inclusion in the study with a single Suna trap hung outside each of seven houses during every experimental night. One MM-X trap was used as a control on the eighth house; this trap was suspended at 15 cm above ground level according to previously established optimal height positioning for this trap [29]. Seven Suna traps were positioned at 15, 30, 45, 60, 75, 90 and 105 cm above ground level. The maximum height was as high as it was possible to suspend a trap before it would have been above the roof level of a house. Trap heights were rotated on a nightly basis so that every trap type + height combination was tested four times at each house – a total of 256 trap nights (32 experimental nights).

Traps were set at dusk (around 18:30 h) and collected at around 07:00 h the following morning. All traps were baited with the MB5 lure [38], as well as CO_2_ produced through fermentation of molasses. Upon returning to the field laboratory, traps were placed in a -20°C freezer to knock down mosquitoes for counting and identification to species-group level on the basis of morphological characteristics [40].

### Statistical analysis

#### ***Suna trap compared with MM-X trap in laboratory and semi-field conditions (dual choice experiments)***

The mean response rate was calculated as the mean proportion (%) of released females captured in either or both traps during each experimental replicate. Mean catch sizes were calculated for each trap type. Independent effects of trap position, temperature or humidity on catch size were tested using a generalized linear model with log link function and negative binomial distribution. Whether the distribution of mosquitoes between the two traps differed from a 1:1 distribution was estimated using a χ^2^ test with two-tailed P-value.

#### ***Suna trap sampling efficacy under semi-field conditions***

Mean catch sizes for the CDC LT, MM-X trap and Suna trap were calculated. The effect of trap type, rainfall, temperature and humidity as independent predictors of the number of mosquitoes caught in a trap were modelled using generalized linear models with a Poisson distribution and log link function. The effect of each predictor was modelled independently (univariate analysis), and predictors with P < 0.1 were included in a multivariable model of trap performance (number of mosquitoes caught in a trap). The association between number of mosquitoes found resting on the interior the walls of the house and each predictor variable was also assessed in the same way.

#### ***Suna trap as an intervention tool – prevention of mosquito house entry***

House entry rates were estimated as mean CDC LT catch sizes and mean number of mosquitoes resting on the interior walls of the house per study night. Ultimately these two values were combined to give a measure of house entry, and mean house entry rates were compared between nights when the Suna trap was suspended outside the house (intervention) and when it was absent (control). Univariate analyses tested for associations between mosquito house entry with intervention status, temperature and humidity. A multivariable GLM with Poisson distribution and log link function was constructed using intervention status and relative humidity as predictors of mosquito house entry.

#### ***Optimal height positioning of the Suna trap in field conditions***

Species composition of female mosquitoes was calculated as the proportion (%) that each species group (*Anopheles gambiae* sensu lato, *Anopheles funestus*, *Culex* species) formed of the total female catch from all traps. Data were combined for unfed, fed and gravid females. Anticipated predictors of catch size (trap type + height, sampling location and night) were tested for an association with catch size using a generalized linear model with log link function. Variables that were significant in univariate analysis (P < 0.1) were included in a multivariable model. Estimated marginal means (EMM) were calculated for each trap type + height position for *An. gambiae* s.l.*, An. funestus* and *Culex* females. All statistical analyses were performed using SPSS statistics version 19 (IBM corporation).

## Results

### Comparing the performance of the Suna trap and MM-X trap in a dual-choice experiment (laboratory and semi-field)

Across 14 experimental replicates, a total of 700 female *An. coluzzii* were released into the behavioural room at Wageningen University. The average temperature was 26.7°C (min 25.0°C, max 28.0°C) and average relative humidity (RH) was 62.3% (min 50%, max 73%).

The mean response rate was 85.7% (±3.0 standard error of the mean). The mean catch in the MM-X trap was 10.4 (±1.2 SE; a mean 20.7% of released females), and the mean catch in the Suna trap was 33.3 (±2.40 SE; a mean 66.0% of released females).

**Figure 2:**
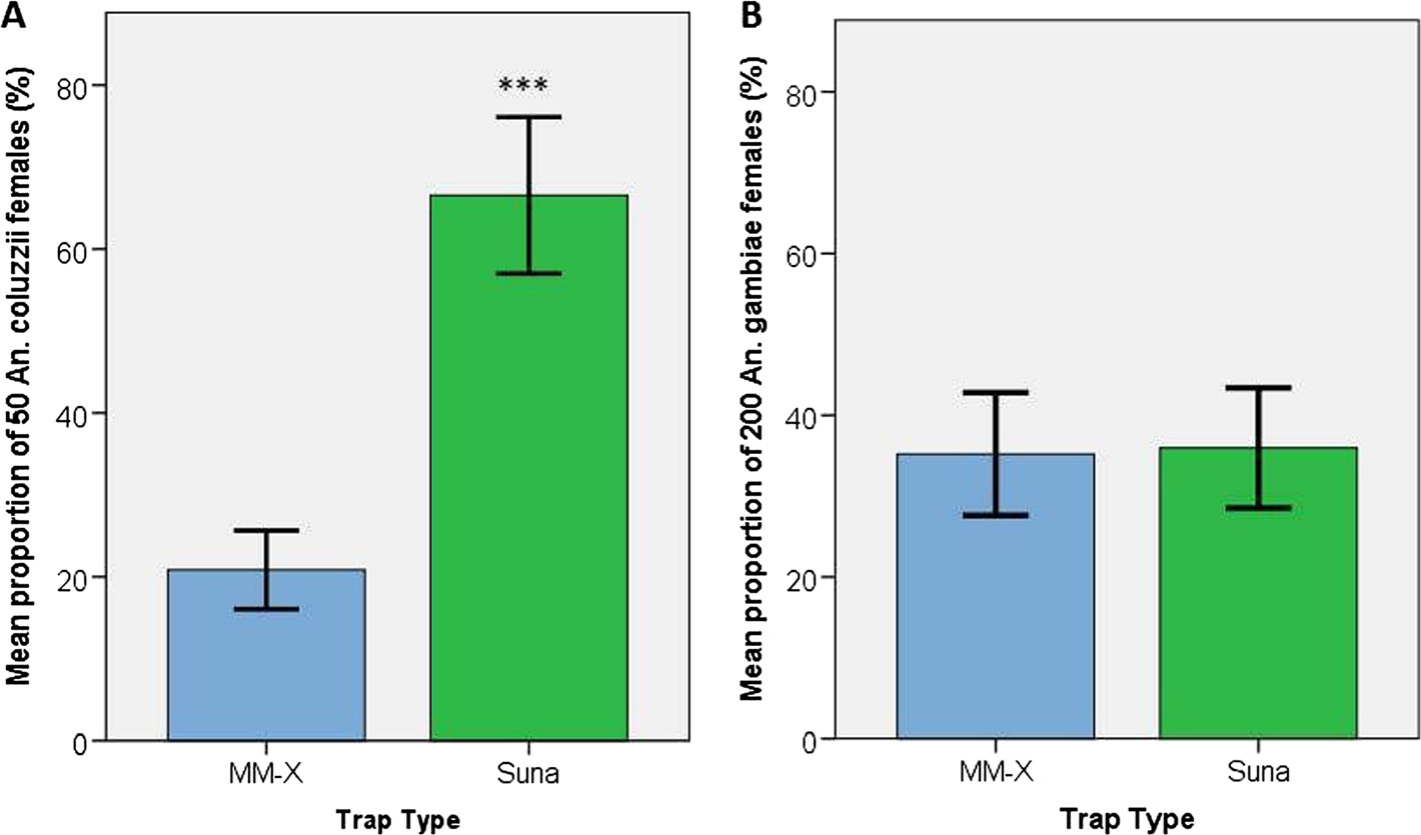
**Mean proportion of females caught in each trap type in A: laboratory conditions (n = 50 females released per replicate), B: semi-field conditions (n = 200 females per replicate).** Error bars indicate ± 2 SEM. ***indicates P < 0.001 for a difference in the distribution of mosquitoes between the two traps.

Under behavioural room conditions there was no evidence for positional bias, and temperature and RH were not linearly associated with catch size. The mean proportion of released mosquitoes caught in the Suna trap was 3.2 times greater than the proportion caught in the MM-X trap (Figure 2). The total Suna trap catch (N = 466 females) differed significantly compared with the catch in the MM-X trap (N = 146 females) (χ^2^ = 167.3, 1 d.f., P < 0.001).

Over the course of 16 nights (32 trap nights) 3,200 *An. gambiae* were released into the screenhouse in Kenya. The average temperature was 24.0°C (min 20.0°C, max 28.4°C) and average RH was 73.0% (min 58.8%, max 89.2%). Both traps functioned normally during all replicates and all data points were included in the analysis.

The mean response rate in the screenhouse was 71.1% (±4.76 SEM). The mean catch in the MM-X trap was 70.4 female *An. gambiae* (±7.6 SEM; 35.2% of released females) while the mean Suna trap catch size was 71.9 females (±7.5 SEM; 35.9% of released females). Catch sizes for both trap types were positively skewed, MM-X trap catch sizes ranged from 31 up to 137 females per night and the Suna trap ranged from 31 to 138 females in a single night. There was no evidence that trap position in the screenhouse had a significant impact on mean mosquito catch sizes. Average nightly temperature and RH were also not found to be associated with catch size.

The mean proportion of released mosquitoes caught in each trap per night was calculated (Figure 2) and the total number of mosquitoes trapped in the Suna trap (N = 1150) compared with the MM-X trap (N = 1126) was not found to differ significantly from a 1:1 distribution (χ^2^ = 0.253, 1 d.f., P = 0.615).

### Assessing the performance of the Suna trap as a monitoring tool – relative sampling efficacy

During each of the two experiments, both of 24 nights duration, 4,800 female *An. gambiae* were released in the MalariaSphere. During the first experiment (CDC LT light off) 49.6% of these females were caught in a trap, and 1.3% were found resting on the walls inside the MalariaSphere house when the experiment ended in the morning. During the second repetition of the experiment (CDC LT illuminated), 46.2% of females were trapped and 2.1% were found resting on the interior walls of the house the following morning. Mosquito flight responses were good, with only 12 females (first series of experiments) and 26 females (second series) remaining in release cups in the morning after experiments. During the course of both experiments all traps functioned normally and climatic conditions were in a range that is normal for this region. All data points were included in the analysis.

In the first series (CDC LT light off), mean catch sizes were greater in the MM-X trap (mean 108.4 ± 6.0 SEM) and the Suna trap (mean 108.8 ± 5.5 SEM) compared with the CDC light trap (mean 80.6 ± 8.4 SEM). The distribution of catch sizes differed between the trap types (see Additional file 3), with the CDC LT having the smallest range of catch sizes. The number of mosquitoes captured was positively skewed for all three types of trap. In the second series (CDC LT illuminated), the mean catch size was greatest in the CDC LT (mean 103.4 ± 3.6 SEM), followed by the Suna trap (mean 95.8 ± 3.5 SEM) with the lowest mean catch in the MM-X trap (mean 77.9 ± 3.1 SEM). Catch sizes for the CDC LT and Suna trap were slightly negatively skewed, whereas MM-X trap catch sizes were slightly positively skewed.

During the first series of experiments there was some indication that temperature and humidity had a small but significant effect on catch size but, as temperature and rainfall both contribute to environmental humidity, in the final analysis only humidity was included as a climatic predictor of catch size. During the second series using the CDC LT with light on, there was no evidence of a univariate association between temperature, humidity and catch size.

**Figure 3:**
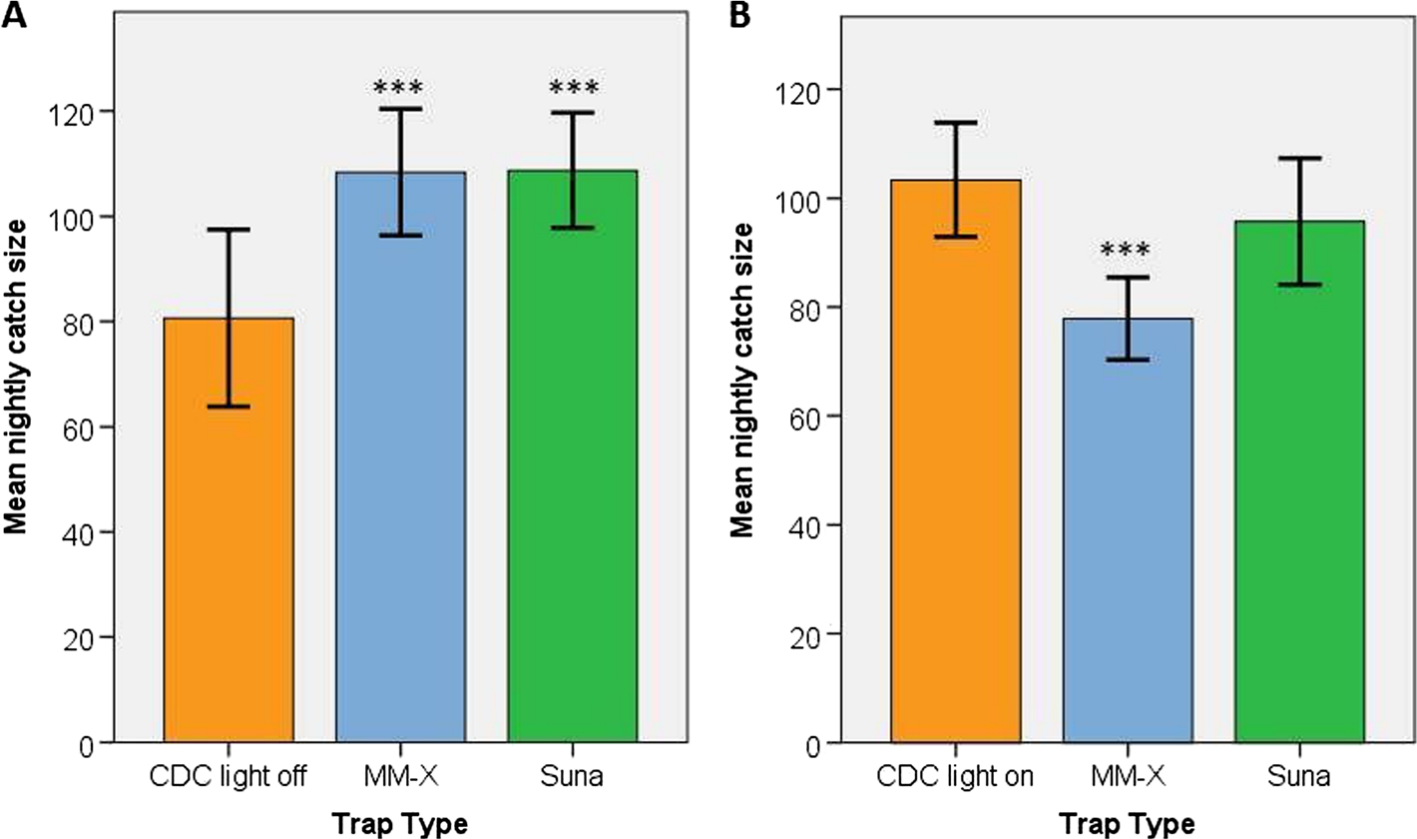
**Mean nightly catch sizes in the A: CDC LT (light off), MM-X trap and Suna trap, B: CDC LT (light on), MM-X trap and Suna trap during experiments in the MalariaSphere.** (Error bars represent ± 2 SEM, N = 8 trap nights for each trap during each series of experiments, n = 200 female *An. gambiae* released during each experimental night). ***indicates P < 0.001 for a difference in catch size, relative to the CDC LT.

After accounting for humidity in the MalariaSphere, there was strong evidence to suggest that female *An. gambiae* were more likely to be trapped using a Suna trap (RR = 1.351, 95% CI: 1.22 – 1.50, P < 0.001) or the MM-X trap (RR = 1.343, 95% CI: 1.21 – 1.49, P < 0.001) compared with a CDC LT with the light off (Figure 3). In the second experiment, the performance of the Suna trap was found to be equivalent to that of an illuminated CDC LT (RR = 0.926, 95% CI: 0.840 – 1.022, P = 0.127), whilst catch sizes in the MM-X trap were significantly lower than in the illuminated CDC LT (RR = 0.753, 95% CI: 0.679 – 0.836, P < 0.001) (Figure 3).

In order to assess whether the number of mosquitoes entering the house, but not caught in a trap, differed according to trap method, the number of females resting on the interior walls of the house in the morning was compared between nights during which each trap type was used (see Table 1). The effect of temperature, rainfall and humidity on indoor resting was also investigated. Univariate analyses indicated that increased humidity was associated with lower indoor resting densities (RR = 0.961, 95% CI: 0.925 – 0.999, P = 0.045). In the second experiment (CDC LT illuminated) there was no evidence of an association between temperature or humidity on indoor resting.

**Table 1:** **Mean indoor resting densities of *An. gambiae *** **females during trap comparison experiments in the MalariaSphere**

**Experiment**	**Trap type**	**Mean indoor resting density (per night)**	**SEM**	**P value**
1	CDC LT light off	3.5	0.7	-
	MM-X	2.3	0.5	0.127
	Suna	2.0	0.5	0.062
2	CDC LT light on	3.0	0.6	-
	MM-X	4.8	0.8	0.078
	Suna	4.6	0.8	0.099

Means in experiment 1 are adjusted for humidity. SEM indicates 1 standard error of the mean, - indicates comparison group for analysis.

Multivariable analysis adjusted for reduced indoor resting with increased humidity in the first experiment, indicated that there was no evidence for a difference in indoor resting with the MM-X trap (RR = 0.636, 95% CI: 0.355 – 1.137, P = 0.127) or Suna trap (RR = 0.559, 95% CI: 0.303 – 1.030, P = 0.062) compared with the unlit CDC LT. In the second experiment, trap type was also not a significant determinant of residual indoor resting (MM-X trap RR = 1.583, 95% CI: 0.950 – 2.639, P = 0.078, Suna trap RR = 1.542, 95% CI: 0.922 – 2.577, P = 0.099).

### The performance of the Suna trap as an intervention tool to reduce mosquito house entry in semi-field conditions

Over the course of 32 nights, 6,400 unfed female *An. gambiae* were released in the MalariaSphere. A total of 3,573 (55.8%) of these females were recaptured in either the Suna trap, the CDC LT or resting on the interior walls of the house. Mosquito flight activity was good with only 31 (0.48%) females remaining in any of the four release cups during the morning after an experiment. As in the previous experiment it was assumed that survival of a female to the following day was unlikely.

During the 32 nights of the experiment, temperature and humidity levels were normal for the time of year (mean nightly temperature 23.7°C, min 19.1°C, max 29.4°C; mean RH 73.3%, min 37.0%, max 99.0%; mean rainfall 5.5 mm, min 0 mm, max 32.7 mm). All traps functioned normally throughout the course of the experiment and all data points were included in the analysis. The distributions of CDC LT catch sizes (as a measure of mosquito house entry) were slightly positively skewed, with a narrower range of catch sizes when the Suna trap was hung outside the house (intervention status), compared with when there was no trap outside the house (control status).

When the Suna trap was not suspended outside the house (control situation) a mean 84.1 females entered the CDC LT inside the house each night (±6.3 SEM, N = 16 trap nights), but when the Suna trap was in place outside the house the mean CDC LT catch reduced to 56.7 females per night (±4.0 SEM).

The mean number of *An. gambiae* found resting inside on the walls of the MalariaSphere house was also reduced when the Suna trap was hung outside the house compared with when no Suna trap was used (control mean 3.56, ± 0.4 SEM, intervention mean 2.25, ± 0.2 SEM).

As both CDC LT catch sizes and indoor resting were reduced by suspending a Suna trap outside the house, these two outcome variables were combined to give a single measure of house entry rate per night (sum of CDC LT catch and indoor resting catch) and this measure was used for subsequent analyses.

**Figure 4:**
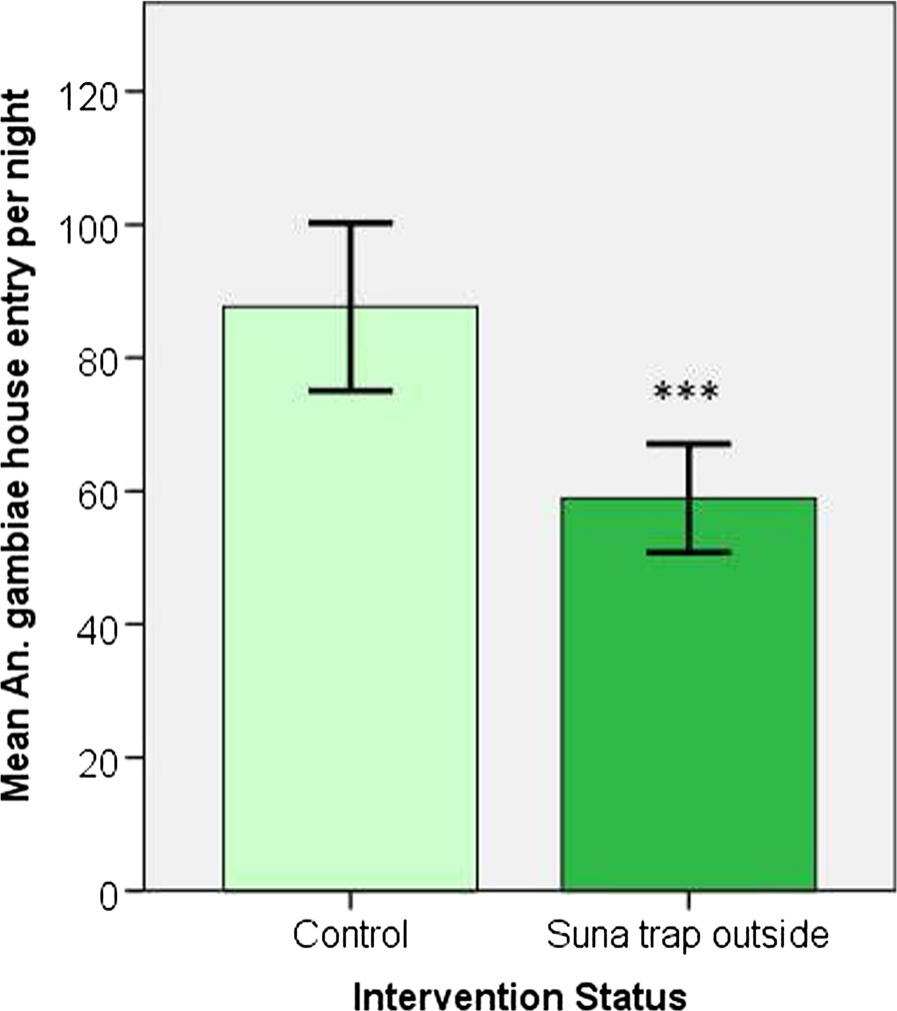
**Mean female *An. gambiae s.s. *house entry per night (N = 16 nights control, 16 intervention, n = 200 females released each night).** *** indicates P < 0.001 for a difference between means using a GLM with Poisson distribution and log link function. Error bars indicate ± 2 SEM.

After adjusting for humidity, there was strong evidence that suspending a Suna trap outside the MalariaSphere house was associated with a 32.8% reduction in *An. gambiae* house entry (CDC LT + indoor resting catch), relative to the control situation where there was no trap positioned outside the house (estimated marginal mean (EMM) house entry for control = 87.7 ± 2.3 SEM, for intervention = 58.9 ± 1.9 SEM; RR = 0.671, 95% CI: 0.618 – 0.729, P < 0.001) (Figure 4).

### Optimal height positioning of the Suna trap under field conditions

Over 256 trap nights a total of 7,620 female and 269 male mosquitoes were captured in traps outside houses in Ahero. *An. gambiae* s.l. formed 41.4% of the total female catch (mean catch per trap per night = 12.3 ± 1.5 SEM), and *An. funestus* group comprised 15.5% of the female catch (mean = 4.6 ± 0.3 SEM). The remainder of the female catch comprised of *Culex* spp (17.0%), *Mansonia* spp (23.9%) and non-identifiable specimens (2.2%).

The house where the trap was positioned was associated with catch size, with mean nightly catch size per house ranging from 5.4 (±1.1 SEM) to 15.7 (±2.9 SEM) *An. gambiae* s.l. (EMM adjusted for trap type + height and house). Study night was also associated with catch size and was included in the multivariable analysis of *An. gambiae* s.l. catch data.

**Figure 5:**
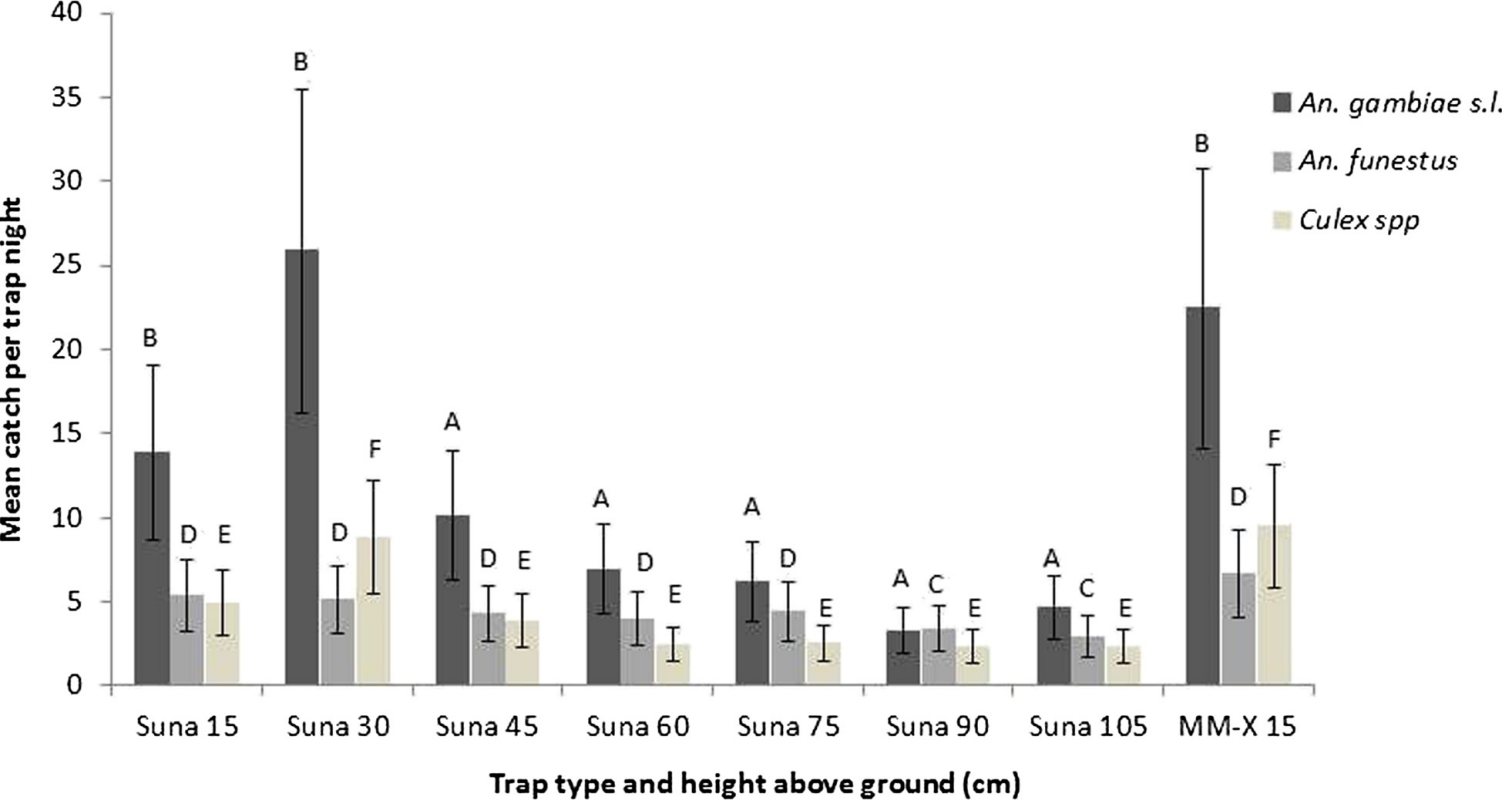
**Estimated marginal mean catch sizes for female mosquitoes caught in Suna traps at varying heights above ground, and in an MM-X trap positioned at 15 cm above the ground, outside houses in the field.** Means are adjusted for day of sampling (*An. gambiae* s.l. only) and house (all species). Error bars represent ± 2 SEM. Different letters above bars indicate significant differences in catch sizes (**A** and **B** for *An. gambiae* s.l., **C** and **D** for *An. funestus*, **E** and **F** for *Culex* spp).

Comparison of catch sizes between traps, adjusting for house and night of sampling in multivariable analysis, revealed that there was no statistically significant difference between *An. gambiae* s.l. catch size in the Suna trap at 15 cm or 30 cm above the ground, relative to the MM-X trap suspended at 15 cm (EMM Suna catch 15 cm = 13.8, RR = 0.617, 95% CI: 0.366 – 1.039, P = 0.069; EMM Suna 30 cm = 25.8, RR = 1.152, 95% CI: 0.682 – 1.949, P = 0.597, EMM MM-X catch = 22.4, ± 4.2 SEM). With each successive 15 cm height increase above 30 cm, catch sizes in the Suna trap decreased and traps suspended with the ventilator opening at 45 cm and higher above the ground were significantly less likely to catch *An. gambiae* s.l. than the MM-X trap at 15 cm (see Figure 5).

After adjusting for house, the greatest mean *An. funestus* catch sizes were in the MM-X trap at 15 cm above ground level (EMM = 6.7 ± 1.3 SEM), but *An. funestus* catch sizes in the Suna trap at 15, 30, 45, 60 and 75 cm were not significantly lower than those of the MM-X trap (Figure 5). At 90 and 105 cm above the ground *An. funestus* catch sizes in the Suna trap were lower than for the MM-X trap at 15 cm above the ground (Suna 90 cm RR = 0.508, 95% CI: 0.293 – 0.883, P = 0.016, Suna 105 cm RR = 0.438, 95% CI: 0.251 – 0.763, P = 0.004). Houses varied in their degree of attraction to *An. funestus*, with adjusted mean catch sizes ranging from 2.9 (±0.6 SEM) to 6.2 (±1.2 SEM) females per night. *Anopheles funestus* catch size was not found to be significantly associated with study night.

After adjusting for house, maximum catch sizes for *Culex* females were obtained using the MM-X trap at 15 cm above the ground (EMM = 9.4, ± 1.8 SEM) and the Suna trap at 30 cm above ground level (EMM = 8.8, ± 1.7 SEM). The difference between these catch sizes was not statistically significant (RR = 0.934, 95% CI: 0.547 – 1.596, P = 0.803). *Culex* catch sizes in the Suna trap at 15, 45, 60, 75, 90 and 105 cm above ground level were all significantly lower than in the MM-X trap at 15 cm above the ground (P = 0.017, P = 0.001, P < 0.001, P < 0.001, P < 0.001, P < 0.001 respectively) (Figure 5). Mean catch sizes by house ranged from 2.1 (±0.46 SEM) up to 9.8 (±1.8 SEM) and study night was not significantly associated with *Culex* catch size.

## Discussion

These results describe for the first time an evaluation of the Suna trap as a tool for trapping host-seeking mosquitoes, and as an intervention tool against *An. gambiae* house entry. It has been demonstrated that, under laboratory and semi-field conditions, the performance of the Suna trap surpasses or is equivalent to that of the MM-X trap. When positioned in an experimental hut in semi-field conditions the Suna trap is more efficacious than an unlit CDC LT, and equivalent in performance to a lit CDC LT. This research has shown that the Suna trap effectively collects mosquitoes outside houses in the field, in numbers that are equivalent to those caught in the MM-X trap. In addition, positioning the Suna trap outside an experimental hut in semi-field conditions, reduces the number of mosquitoes entering a hut occupied by a human.

Under laboratory conditions, the enhanced performance of the Suna trap, relative to the MM-X trap, could be explained by the wider plume of odour dispersed by the Suna trap at point of origin, which probably attracts more mosquitoes than the MM-X trap. In addition, it is expected that, once in close proximity to the Suna trap, mosquitoes are more likely to be sucked inside as the airflow of the Suna trap is greater than that of the MM-X trap. Under semi-field and field conditions the trapping efficacy of the Suna trap and MM-X trap were equal. It is assumed that natural dispersal of the air plume, and its odorous components, combined with the constant shifting direction of the prevailing wind [41], create similar odour fronts for the mosquitoes to respond to [42], and that the effect of the wider point of origin odour plume in the Suna trap compared with the MM-X trap, is reduced. The mosquitoes used for experiments in the laboratory were *An. coluzzii*, while those used in semi-field experiments were *An. gambiae s.s.*, but as the difference between forms M and S occurs in a small region of the X chromosome and both forms are anthropophilic it is not expected that this would contribute to a difference in response to the Suna trap.

Despite the equivalent performance of the two traps under field conditions, the MM-X trap was developed as a research tool and is currently not commercially available. The Suna trap provides a good alternative for the MM-X trap. The construction of this trap is technologically much simpler than that of the MM-X trap, rendering it a cheaper, but equally effective tool for sampling mosquitoes.

During the course of this study the relative performance of the CDC LT was found to be higher when the lamp was illuminated compared with when it was not illuminated. The amount by which the performance was increased could not be directly quantified as the two experiments were conducted nine months apart, but this finding is in line with those of Costantini and Mweresa who also found that illuminated light traps had greater catch sizes compared with unlit traps [23,43]. In comparison with the CDC LT, both lit and unlit, the Suna trap formed a good tool for sampling mosquitoes from inside a house occupied by a human sleeping under a bed net. As it has previously been demonstrated that the CDC LT is not an effective tool for monitoring outdoor biting mosquitoes [23], the traps were not compared outdoors.

In some settings the CDC LT has been considered as a proxy for the HLC, with catch sizes from the two methods being proportional to one another [24]. It can be inferred from the results described here that the Suna trap and MM-X trap baited with the MB5 blend of odours and CO_2_, provide an effective alternative for the CDC LT (beside a human-occupied bed net) and the relative catch size could, therefore, also be related to that of the HLC for *An. gambiae*.

The use of odour-baited traps, such as the Suna trap, carries an advantage over the more traditionally used CDC LT when used as a tool for estimating changes in possible human biting rate and background mosquito population density. Host-seeking mosquitoes are specifically attracted to the trap because the odours emitted mimic those of a human host [20,31,44]. Additionally the Suna trap is an effective tool for outdoor use, which does not apply in the case of the CDC LT. By using a standardized odour bait, the Suna trap is expected to exhibit a consistent relative sampling efficacy both indoors and outdoors throughout the night, as under stable environmental conditions the odours are released at a constant rate. Thus, this new trap holds great potential to form a replacement for the HLC, which has significant associated health risks and is subject to inter-individual variation in attractiveness to mosquitoes [45], as well as ability of volunteers to aspirate mosquitoes during a HLC [46].

As well as establishing that Suna traps are an effective means of sampling mosquitoes that entered a hut under semi-field conditions, it was shown that the Suna trap forms an excellent tool for sampling mosquitoes outside houses in a rice-growing area of western Kenya. The majority of anopheline mosquitoes sampled during this study belonged to the *An. gambiae* complex, and previous studies conducted in the same area identified all, or 99%, of *An. gambiae* s.l. collected from this village as *Anopheles arabiensis*[43,47]. This indicates that the Suna trap baited with the five-component blend of odours and CO_2_ is an effective tool for sampling this member of the *An. gambiae* complex, as well as *An.coluzzii* and *An. gambiae s.s*. As recent studies have demonstrated shifts towards outdoor biting among populations of *An. gambiae* s.l. and *An. funestus* in Tanzania and Equatorial Guinea [48,49], and there is a growing awareness that many malaria vectors are exophilic [50], it becomes increasingly important to be able to monitor these vector populations outside houses. The Suna trap could form an ideal tool for this purpose.

During this field study to evaluate the optimal hanging height of Suna traps positioned outside houses, buildings of the same construction, in similar locations, and with equivalent occupancy rates and reported bed net usage were selected. Despite this, there was significant house-to-house variation in catch sizes. It is recommended that mosquito monitoring programmes sample host-seeking mosquitoes in a range of different locations in order to make accurate measures of population density. Suna traps provide the advantage that many traps, baited with the same odour, can be operated simultaneously with relatively low labour costs. There is no need for an operator or human volunteer to be present during the nocturnal catching period.

As well as demonstrating that the Suna trap could be used for mosquito monitoring inside a house, the semi-field testing environment was used to demonstrate that suspending a Suna trap outside a house with a single occupant underneath a bed net could reduce *An. gambiae* house entry rates by 32.8%, compared to the situation where a bed net is the only form of personal protection against mosquito bites. This estimate of odour-baited trap efficacy should be integrated into models, such as the one developed by Okumu and others [35], which could be used to estimate the potential efficacy of mass-mosquito trapping as an intervention against malaria control when used in combination with ITNs.

Similar to these findings, Smallegange *et al.* reported reductions in *An. gambiae* house entry of almost 80% when using odour-baited MM-X traps with yeast-produced CO_2_ in the MalariaSphere [51]. In contrast, Jawara and others [29] did not observe any reduction in house entry when using odour-baited MM-X traps outside experimental huts in the Gambia. Differences in environmental conditions, trap type and composition of the odour bait may explain these seemingly contrasting findings.

Other interventions against mosquito house entry, such as burning repellent plants, adding ceilings to houses or closure of the eaves, have reduced *An. arabiensis* and *An. gambiae* house entry rates by more than 70% [52-54], but these methods presumably divert mosquito bites away from members of the protected house towards unprotected members of the community. The Suna trap collects mosquitoes that are flying around the peri-domestic environment, thus the protective effect is expected to be communal. An additional advantage of odour-baited traps for vector control, compared with existing approaches such as insecticide-treated bed nets and indoor residual spraying, is the absence of an insecticide in the traps. An intervention based on mass-trapping of mosquitoes will not contribute to already-increasing levels of insecticide resistance that threaten the long-term success of other vector control techniques. Furthermore, when used outside a house, the Suna trap provides a means of targeting exophagic mosquitoes and outdoor malaria transmission, which has been recommended as an essential step towards eventual elimination of malaria where ITNs, IRS and case management alone are insufficient [50,55-57].

The SolarMal project [58] on Rusinga Island, western Kenya, aims to evaluate for the first time whether the mass deployment of odour-baited Suna traps can be used to control malaria in an area where bed nets and case management are the existing mainstays of malaria control. These semi-field investigations into reductions in mosquito house entry that can be achieved by suspending the Suna trap outside a traditional house yielded promising results and the next stage of evaluating the Suna trap as a tool for malaria control in the field is eagerly anticipated.

## Conclusions

The sampling efficacy of the Suna trap equals that of the illuminated CDC LT, with the added advantage that it does not require the presence of a human volunteer and that it can also be used to sample mosquitoes outdoors. As the trap uses a standardized odour bait, the variation in catches caused by differential attractiveness of HLC volunteers, or human-baited CDC LTs, is avoided, thus enhancing opportunities for routine and objective mosquito monitoring. When hung outside an experimental house, entry by *An. gambiae* is significantly reduced, suggesting that daily removal trapping by the Suna trap could provide a new tool for controlling malaria transmission in and around the peri-domestic environment in villages of sub-Saharan Africa.

## Abbreviations

CDC LT: Centers for Disease Control light trap; EIR: entomological inoculation rate; EMM: estimated marginal mean; IRS: indoor residual spraying; ITN: insecticide-treated bed net; MM-X trap: Mosquito Magnet-X^®^ trap; RH: relative humidity; RR: relative rate; SEM: standard error of the mean.

## Competing interests

AH, BO, AK, CKM, PO, MG, AR, WRM and WT declare that they have no competing interests. The association of MG and AR with Biogents did not affect the decision to publish the data presented here and these authors were not involved in the experimental study design.

## Authors’ contributions

AH, WT, WRM, MG and AR designed and developed the Suna trap. AH, BO, AK, CKM and PO designed and carried out the laboratory, semi-field and field experiments. AH and WT analysed the data. AH, WT and WRM drafted the manuscript. All authors read and approved the final manuscript before submission.

## Supplementary Material

Additional file 1 **A; Layout of the behavioural room at Wageningen University, The Netherlands. B;** Layout of the screenhouse in Kenya. In both diagrams the dashed line represents net screening, A and C refer to the positions of two traps. During a dual-choice experiment the position of each trap is alternated before every experimental replicate. Click here for file

Additional file 2 **Layout of the MalariaSphere used during trap comparison studies, and in studies to estimate reductions in mosquito house entry when using a Suna trap.** During the trap comparison study, traps were suspended inside the house at position Y. During the Suna trap as an intervention study the Suna trap was suspended outside the house at position X on alternate nights. Click here for file

Additional file 3 **A: Boxplot showing the minimum, first quartile, median, third quartile and maximum catch size for A: the CDC LT (light off), MM-X trap and Suna trap (N = 8 trap nights for each type of trap). B:** the CDC LT (light on), MM-X trap and Suna trap (N = 8 trap nights for each type of trap. Click here for file
