# Supplementary figures and images for: Development and optimization of the Suna trap as a tool for mosquito monitoring and control
Source: Malar J. 2014 Jul 7;13:257. doi: 10.1186/1475-2875-13-257 (PMC4105527; doi:10.1186/1475-2875-13-257)

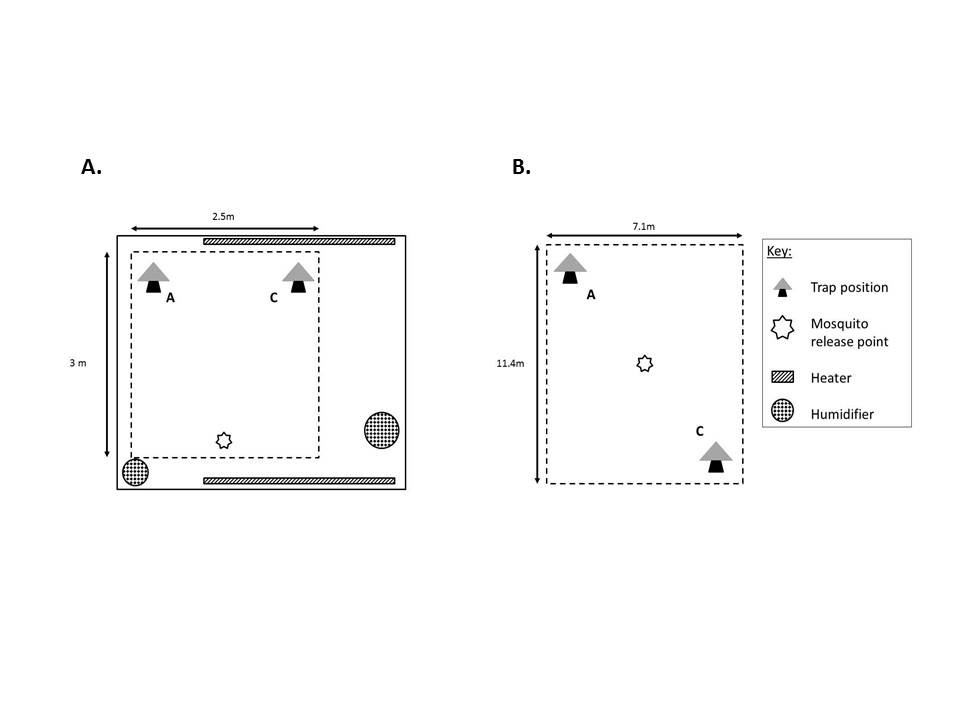

Supplement: Additional file 1 — A; Layout of the behavioural room at Wageningen University, The Netherlands. B; Layout of the screenhouse in Kenya. In both diagrams the dashed line represents net screening, A and C refer to the positions of two traps. During a dual-choice experiment the position of each trap is alternated before every experimental replicate. [file 1475-2875-13-257-S1.jpeg]

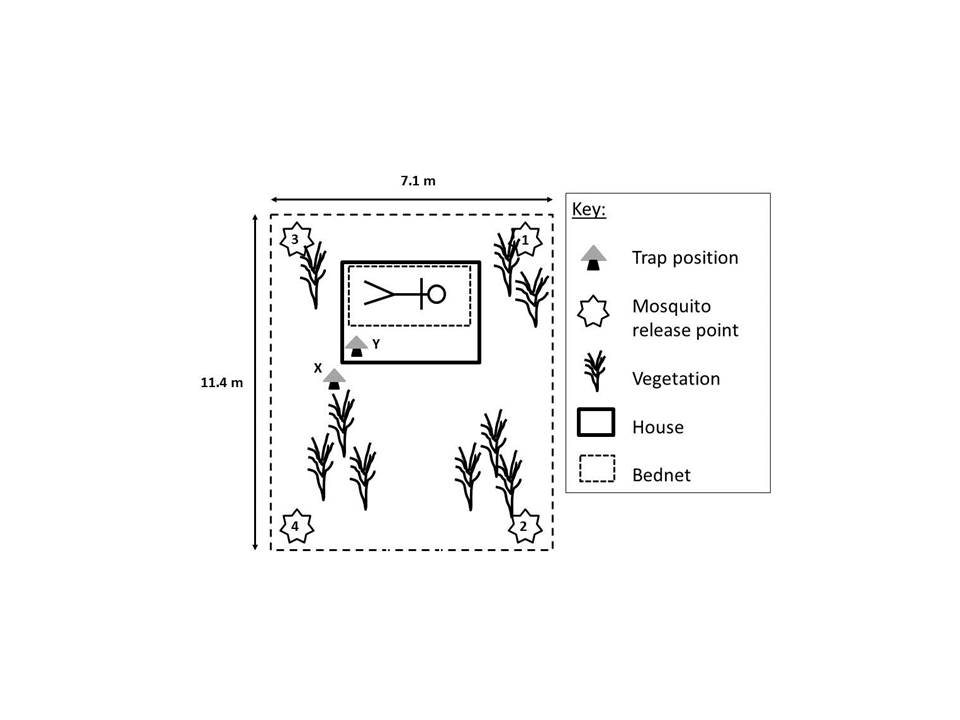

Supplement: Additional file 2 — Layout of the MalariaSphere used during trap comparison studies, and in studies to estimate reductions in mosquito house entry when using a Suna trap. During the trap comparison study, traps were suspended inside the house at position Y. During the Suna trap as an intervention study the Suna trap was suspended outside the house at position X on alternate nights. [file 1475-2875-13-257-S2.jpeg]

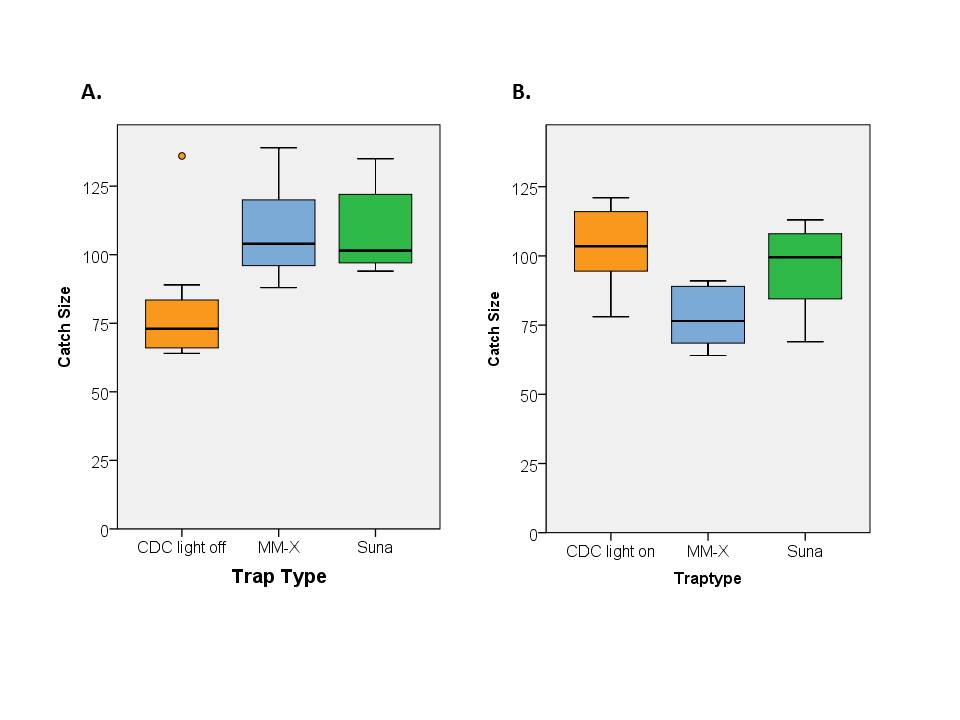

Supplement: Additional file 3 — A: Boxplot showing the minimum, first quartile, median, third quartile and maximum catch size for A: the CDC LT (light off), MM-X trap and Suna trap (N = 8 trap nights for each type of trap). B: the CDC LT (light on), MM-X trap and Suna trap (N = 8 trap nights for each type of trap. [file 1475-2875-13-257-S3.jpeg]
